# BioTM buzz

**DOI:** 10.1002/btm2.10080

**Published:** 2017-10-17

**Authors:** Aaron C. Anselmo

**Affiliations:** ^1^ Division of Pharmacoengineering and Molecular Pharmaceutics, Eshelman School of Pharmacy University of North Carolina at Chapel Hill Chapel Hill NC 27599 USA

## Volume 2, Issue 3

### Patient‐Friendly Peptide Delivery

Reliable delivery of peptides through minimally invasive routes is an unaddressed challenge that could dramatically change clinical care. Currently, the majority of peptides are delivered through injections, and as such there is significant need for patient‐friendly delivery systems. In this issue of *Bioengineering & Translational Medicine*, engineers from Nanyang Technological University in Singapore describe a transdermal technology that enables the loading and delivery of therapeutic peptides for local treatment of keloid scars. The authors show that by using a strategy to swell poly(ethylene glycol) diacrylate (PEGDA)‐based microneedles, a gap junction inhibiting peptide, Gap 26, could be loaded (over 50% loading efficiency) into the microneedles without compromising its potency. Gap 26 is used to suppress intercellular communication between keloid fibroblasts and subsequently limit scar formation; the authors show that microneedles loaded with Gap 26 were able to limit collagen expression by over 45% in an ex vivo scar model. The polymer parameters, such as the swelling ratio and mesh size, of the microneedles could be controlled through regulating UV crosslinking and may provide a means to better control loading and release of other peptides or even larger molecules (e.g., antibodies). Overall, this study addresses challenges related to loading, stability, and subsequent delivery of peptide‐based drugs through the transdermal route.

DOI: 10.1002/btm2.10070

### Biologics for Stem Cell Expansion

Stem‐cell based therapies have immense potential to treat complex diseases that require significant tissue regeneration, such as osteoarthritis. For osteoarthritis, the current clinical care focuses on symptom treatment and thus there is a significant need for therapies that can treat and repair damaged tissues. While stems cells are widely investigated in clinical trials, one of the major hurdles facing their translation into a clinical therapy is the expansion and cultivation of these adult stem cells. In this issue of *Bioengineering & Translational Medicine*, the Nidhi Bhutani lab in the Department of Orthopaedic Surgery at Stanford University School of Medicine, report on the use of Collagen VI as a supplemental biologic for the minimally invasive expansion of bone marrow‐derived mesenchymal stem cells (BM‐MSC) as a means to address issues related to in vitro and/or ex vivo stem cell expansion that result in changes to stem cell properties and subsequently their efficacy. In this paper, Collagen VI treatment led to over twofold increase in BM‐MSC proliferation as compared to untreated controls after 4 days. Furthermore, the authors confirmed that the Collagen VI‐treated BM‐MSC retained both their stem cell phenotype and their chondrogenic differentiation potential, which is indicative of their ability to engineer cartilage. The success and translation of stem cell therapies relies on culture methods that can preserve their natural properties while simultaneously enabling scaled‐production; here, the authors show how these challenges can potentially be addressed through the addition of key biologics to stem cell cultures.

DOI: 10.1002/btm2.10078

## Recent literature

### User‐Defined Synthetic Vascular Systems

A research article from the Cole A. DeForest lab from the Department of Chemical Engineering at the University of Washington details a novel approach for the generation of 3D vascular networks that both houses stromal cells and endothelial cells. Importantly, this fabrication method addresses challenges related to control over scaffold size, resolution, and patterning. The approach described here allows for this level of control over vascular organization, which is required to support dense, metabolically active tissue. Vascular channels (ranging in cross sectional sizes from 500 by 500 microns down to 10 by 10 microns) were fabricated via a programmable photodegradation approach. This approach enabled the fabrication of systems that mimicked natural vascular systems both in terms of size, geometry, and cellular makeup through the inclusion of both endothelial cells and stromal cell. These systems afford the freedom of modular design with user‐end control over polymer choice, cellular constituents, and vascular geometry. Broadly, this approach can provide a technological foundation for organ‐on‐chip systems that can be used to study clinically relevant drug–host tissue interactions and also understand basic cell function and biology in relation to other tissues.

*Arakawa et al., Adv Mater. 2017; doi:* 10.1002/adma.201703156

